# Young adulthood body mass index, adult weight gain and breast cancer risk: the PROCAS Study (United Kingdom)

**DOI:** 10.1038/s41416-020-0807-9

**Published:** 2020-03-23

**Authors:** Andrew G. Renehan, Mary Pegington, Michelle N. Harvie, Matthew Sperrin, Susan M. Astley, Adam R. Brentnall, Anthony Howell, Jack Cuzick, D. Gareth Evans

**Affiliations:** 1 Manchester Cancer Research Centre and NIHR Manchester Biomedical Research Centre, Manchester, UK; 2 Division of Cancer Sciences, School of Medical Sciences, Faculty of Biology, Medicine and Health, University of Manchester, Manchester, UK; 3 Prevent Breast Cancer, Nightingale Breast Screening Centre, University Hospital of South Manchester, Manchester, UK; 4 MRC Health eResearch Centre (HeRC), Division of Informatics, Imaging and Data Sciences, School of Health Sciences, Faculty of Biology, Medicine and Health, University of Manchester, Manchester, UK; 5 Centre for Imaging Science, Division of Informatics, Imaging and Data Science, Faculty of Biology, Medicine and Health, University of Manchester, Oxford Road, Manchester, UK; 6 The University of Manchester, Manchester Academic Health Science Centre, University Hospital of South Manchester, Manchester, UK; 7 Centre for Cancer Prevention, Wolfson Institute of Preventive Medicine, Queen Mary University of London, London, UK; 8 Genomic Medicine, Manchester Academic Health Sciences Centre, University of Manchester and Central Manchester Foundation Trust, Manchester, UK

**Keywords:** Risk factors, Oncology

## Abstract

**Background:**

We tested the hypothesis that body mass index (BMI) aged 20 years modifies the association of adult weight gain and breast cancer risk.

**Methods:**

We recruited women (aged 47–73 years) into the PROCAS (Predicting Risk Of Cancer At Screening; Manchester, UK: 2009–2013) Study. In 47,042 women, we determined BMI at baseline and (by recall) at age 20 years, and derived weight changes. We estimated hazard ratios (HRs) and 95% confidence intervals (CIs) for new breast cancer using Cox models and explored relationships between BMI aged 20 years, subsequent weight changes and breast cancer risk.

**Results:**

With median follow-up of 5.6 years, 1142 breast cancers (post-menopausal at entry: 829) occurred. Among post-menopausal women at entry, BMI aged 20 years was inversely associated [HR per SD: 0.87 (95% CI: 0.79–0.95)], while absolute weight gain was associated with breast cancer [HR per SD:1.23 (95% CI: 1.14–1.32)]. For post-menopausal women who had a recall BMI aged 20 years <23.4 kg/m^2^ (75th percentile), absolute weight gain was associated with breast cancer [HR per SD: 1.31 (95% CIs: 1.21–1.42)], but there were no associations for women with a recall BMI aged 20 years of >23.4 kg/m^2^ (*P*_interaction_ values <0.05).

**Conclusions:**

Adult weight gain increased post-menopausal breast cancer risk only among women who were <23.4 kg/m^2^ aged 20 years.

## Background

Body mass index (BMI), as an approximation of general body fatness, is positively associated with risk of post-menopausal breast cancer, yet inversely associated with pre-menopausal breast cancer.^1,2^ Young adulthood (typically aged 18–21 years) BMI, either captured by recall or directly measured, is inversely associated with subsequent risk of both post-menopausal and pre-menopausal breast cancer risk.^3^ This seems paradoxical and might have several explanations. Childhood and early adulthood adiposity may mediate effects on subsequent breast cancer risk through later changes in adipose tissue, for example, weight gain, and downstream hormonal changes. This hypothesis will be the focus of this paper. Additionally, it has recently been appreciated that childhood and early adulthood adiposity might mediate effects on mammary carcinogenesis through mammographic density.^4^

Two recent meta-analyses^5,6^ of prospective studies concluded that adult weight gain (expressed as absolute weight change) is positively associated with post-menopausal breast cancer risk, but not for pre-menopausal breast cancer. Weight gained in adolescence is mainly a combination of gains in muscle mass and adipose tissue, which in women, is distributed primarily on the hips and thighs (pear shaped).^7^ Later in adulthood, most weight gain is through adipose accumulation, preferentially around the waist. In turn, this adipose distribution is associated with adverse metabolic phenotypes and insulin resistance, and might be detrimental for later cancer risk.^8^ Thus, a hypothesis emerges that the absence of excess body fat in late adolescence or early adulthood increases risk of breast cancer, since most of the excess body fatness gained by these women during later adulthood might be metabolically ‘bad’ adipose tissue. This pathophysiology might not apply to women in young adulthood who already have accumulated excess adiposity.

The above meta-analyses covered studies up to 2014^6^ and 2015,^5^ respectively. From these, there were nine cohort studies that reported BMI or weight at ages 18 to 21 years, weight change and breast cancer risk (Supplementary Table S1).^9–17^ Seven studies adjusted for young adulthood BMI or weight. A Nurses’ Health Study analysis (2006),^12^ limited to post-menopausal women, specifically addressed whether the associations of breast cancer risk from weight gain differed by BMI strata at age 18 years, and found stronger associations for lower BMI strata at age 18 years. An updated analysis from the Nurses’ Health Study (2017) reported persistent inverse associations for weight at age 18 years for both pre- and post-menopausal breast cancers, independent of weight gain.^18^

Here, we tested the hypothesis that BMI aged 20 years modifies the effect of the association of adult weight gain and breast cancer risk. We stratified new breast cancer a priori by menopausal status.

## Methods

### Population

The Predicting Risk Of Cancer At Screening (PROCAS) study has been described in detail elsewhere.^19–21^ Between October 2009 and June 2015, 131,373 women aged 46–73 years were, in 15 screening areas across Greater Manchester, United Kingdom, invited for routine three-yearly mammographic screening, were mailed information, a consent form and a two-page questionnaire to elicit information about family history, hormonal and lifestyle risk factors (https://www.ncbi.nlm.nih.gov/books/NBK379485/?report=reader). A total of 57,902 women agreed to enter PROCAS.

### BMI measurements and other variables

Cohort entry BMI was calculated from separately reported weight and height and categorised as follows: low normal weight (18.5–22.4 kg/m^2^), high normal weight (22.5–24.9 kg/m^2^), overweight (25.0–29.9 kg/m^2^) and obese (≥30.0 kg/m^2^).

Participants recalled weight at 20 years of age, and BMI for age 20 years was calculated using the cohort entry reported height. We evaluated the validity of assessing BMI at 20 years of age using recalled weight by comparing mean recall BMI distributions against age- and sex-specific BMI distributions in contemporaneous populations (Supplementary Table S2).^22^ We calculated average absolute weight gain (in kg) from age 20 years to cohort entry; relative weight gain (percentage weight change compared with recall weight aged 20 years); and absolute change per year (kg per year).

We included the following other variables: age at cohort entry, height, age at menarche, race (White, Asian, Black, Jewish, Others, missing), hormonal replacement therapy (HRT: ever/never user), statin (ever/never user), alcohol use (any/no/missing: alcohol consumption was not available), any exercise (any/no/missing: exercise quantity was not available), number of children, age at first pregnancy, hysterectomy, number of ovaries removed, menopausal status (post-menopausal versus pre- and peri-menopausal) defined by Phipps et al.^23^ Women were considered post-menopausal if they reported one or more of the following criteria: natural menopause, surgical menopause involving bilateral oophorectomy; or current use of HRT; pre-menopausal if they self-reported continued menstrual periods or current use of hormonal birth control; and peri-menopausal if they did not meet these criteria and were unsure whether their periods had stopped.

Inclusion for the analysis required full data on BMI at study entry, recall BMI at age 20 years and menopausal status.

### New breast cancer

The primary outcome was diagnosis of a new breast cancer [International Classification of Diseases, Tenth revision, codes C50/D05: invasive breast cancer/ductal carcinoma in situ (DCIS)] from entry screen onwards, as identified through the National Health Service Breast Screening Programme system and the Somerset and North West Cancer Intelligence services. We categorised breast cancer as prevalent (i.e. screen detected at entry) if a tumour was diagnosed within 100 days after initial study entry; the remainder as incident (i.e. interval and subsequent screen detected). While all women were screened, there might be a detection bias across the BMI range—for example, excess body fatness might mask early-stage breast cancer (even on mammography). Thus, we assessed stage presentation across the BMI distribution by capturing T-stage and N-stage according to AJCC 7th Edition.^24^

### Statistical analysis

We tested for trends in baseline characteristics across recall BMI categories at age 20 years using Cuzick’s tests for continuous and Cochrane–Armitage tests for categorical data. We tabulated Pearson’s correlations for the anthropometric parameters.

For time-to-event analyses, we expressed risk in absolute terms per 1000 person years and then estimated hazard ratios (HRs) and 95% confidence intervals (CIs) using Cox models. The timescale was attained age at cohort entry. To standardise our results, we expressed risk estimates per standard deviation (SD) for each anthropometric distribution (as we have done elsewhere^25^). To directly address our hypothesis, we explored relationships between BMI at age 20 years (as a strata) and subsequent weight changes, selecting post hoc the 75th percentile of the BMI aged 20 years distribution (23.4 kg/m^2^) as a cut-off. We justified this cut-off (rather than the median of 21.7 kg/m^2^) on three grounds: (i) using a method similar to that reported by New et al.,^26^ a data-driven exploration revealed potential pivots in risk for BMI values between 23.0 and 24.0 kg/m^2^; (ii) this cut-off was a good predictor for later weight gain (see Results); and (iii) by stratifying the population into a quarter versus three-quarters, there are potential opportunities for targeted public health strategies.

The validity of the proportional hazards assumption was tested using Schoenfeld residuals and visualisation of Kaplan–Meier curves. We used a likelihood ratio test to calculate *P* values, including those for interactions in global models.

We performed sensitivity analyses testing assumptions of our models, separately excluding DCIS and prevalent breast cancers. In the models of associations of BMI at age 20 years with breast cancer, we adjusted for BMI at cohort entry to partially account for unmeasured confounding from a long-term adiposity status during adulthood. We further explored the possibility of selection bias by obesity status (survivor bias), where obese women, who did not develop breast cancer by cohort entry were less susceptible, by testing an interaction term between exposure and age at entry after splitting the timescale into refined age categories. For all analyses, we used STATA (version 15, Stata Corp., TX, USA).

## Results

### Flow diagram

**Fig. 1:**
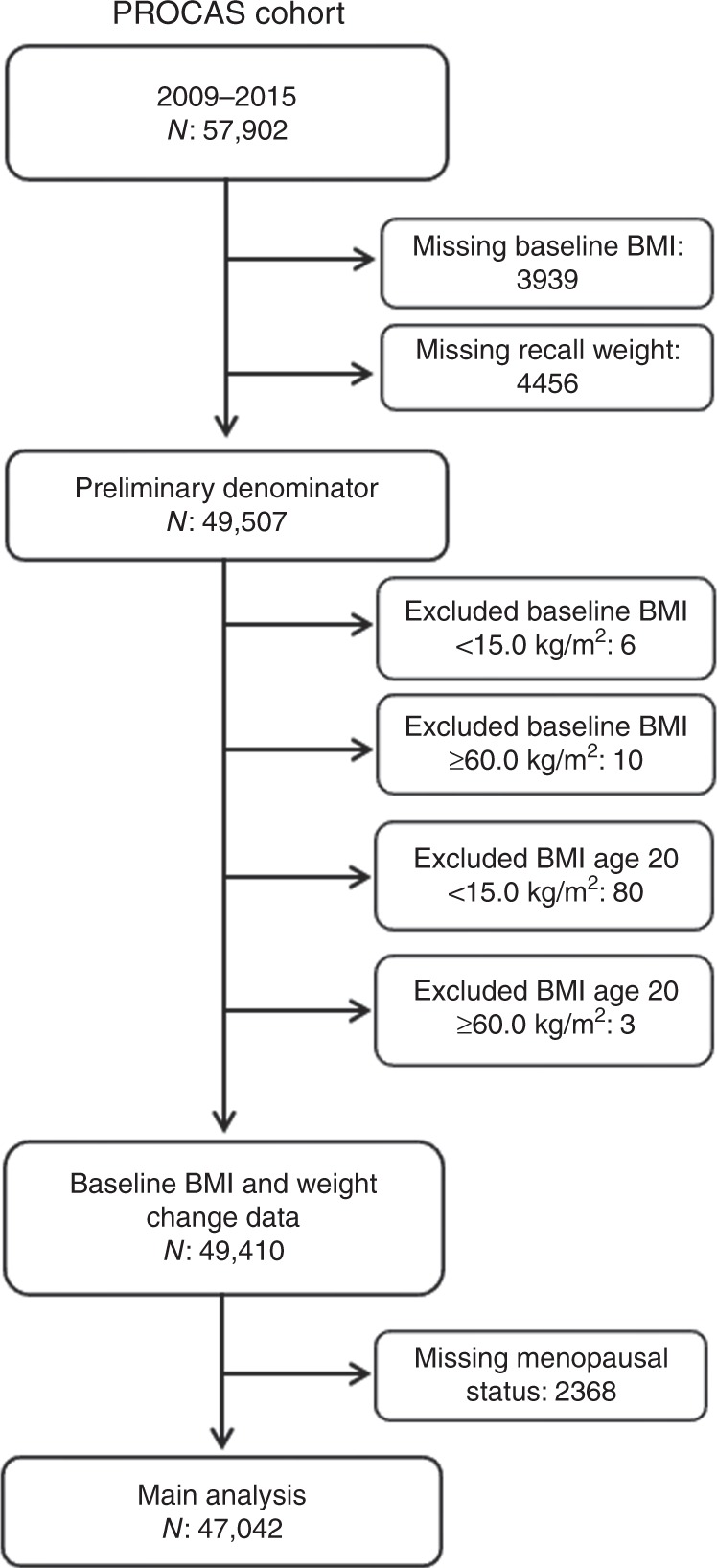
Flow diagram. Number of women in cohirt cohort to those meeting criteria for inclusion in analysis.

From the original cohort of 57,902 women, there were 49,410 women with determined BMI, within the range 15.0–60.0 kg/m^2^, both at cohort entry and recall at age 20 years (Fig. 1). Menopausal status was missing in a further 2368 women (detailed in Supplementary Table S3). Thus, the denominator for the main analysis was 47,042 women.

### Baseline characteristics

The baseline characteristics according to categories of BMI aged 20 years are shown in Table 1. While 92% of the women were Caucasian, this proportion was lower among the underweight BMI aged 20 years category (*P* < 0.001). The following were noted with increasing BMI aged 20 years: decreasing median age at study entry, proportion who were menopausal, and current users of HRT (all *P* < 0.001); any type of oophorectomy (*P* = 0.002); decreasing height (*P* < 0.001); higher proportion ever user of statins (*P* < 0.001); lower use of alcohol (*P* < 0.001); lower median units of any exercise per week (*P* < 0.001); lower median age at menarche (*P* < 0.001) and first pregnancy (*P* < 0.001); and lower proportion who were parous (*P* < 0.001). BMI aged 20 years was correlated with BMI at cohort entry, but not correlated with weight changes (Supplementary Table S4).

**Table 1:** Baseline characteristics in 47,042 women in PROCAS (2009–2015).

	BMI at age 20 years (kg/m^2^)						P value
	Total	<18.5	18.5–22.4	22.5–24.9	25.0–29.9	≥30	
Number of women	47,042	3409	26,065	11,525	4903	1140	
Ethnicity							
White	43,343 (92)	3023 (87)	24,003 (92)	1070 (93)	4549 (93)	1061 (93)	
Asian	550 (1)	107 (3)	319 (1)	83 (1)	32 (1)	9 (1)	
Black	321 (1)	30 (1)	170 (1)	71 (1)	40 (1)	10 (1)	
Jewish	433 (1)	32 (1)	241 (1)	101 (1)	51 (1)	8 (1)	
Other	744 (2)	82 (2)	401 (2)	172 (1)	72 (1)	17 (1)	
Missing	1651 (4)	135 (4)	931 (4)	391 (3)	159 (3)	35 (3)	<0.001^a^
At study entry characteristics							
Median age at study entry (IQR)	57.9 (52.1–64.1)	57.7 (52.1–64.0)	57.9 (52.2–64.0)	58.4 (52.3–64.5)	57.6 (51.9–63.7)	55.6 (51.2–61.7)	<0.001^b ^
Median BMI at study entry (IQR)	26.4 (23.5–30.1)	23.5 (21.1–26.2)	25.0 (22.9–28.0)	28.2 (25.6–31.7)	31.7 (28.2–35.7)	36.8 (30.9–41.3)	<0.001^b^
Mean height (SD)	1.62 (0.06)	1.64 (0.07)	1.62 (0.06)	1.61 (0.06)	1.60 (0.07)	1.61 (0.07)	<0.001^b^
Menopausal status							
Pre-menopausal	5722 (12)	375 (10)	3140 (12)	1363 (11)	658 (13)	186 (15)	
Peri-menopausal	9042 (18)	685 (19)	5064 (19)	2123 (18)	919 (18)	251 (20)	
Post-menopausal	32,278 (65)	2349 (66)	17,861 (65)	8039 (66)	3326 (64)	703 (57)	<0.001^c^
Median age at menopause (IQR)	50 (46–51)	49 (45–51)	50 (46–51)	50 (46–52)	49 (45–51)	49 (44–51)	0.488
Current HRT							
No	29,052 (62)	2013 (59)	15,929 (61)	7138 (62)	3183 (65)	789 (69)	
Yes	17,990 (38)	1396 (41)	10136 (39)	4387 (38)	1720 (35)	351 (31)	<0.001^d^
Hysterectomy							
No	35,421 (75)	2577 (76)	19,914 (76)	8552 (74)	3548 (72)	830 (73)	
Yes	11,621 (25)	832 (24)	6151 (24)	2973 (26)	1355 (28)	310 (27)	<0.001^d^
Oophorectomy							
No	41,167 (88)	2971 (87)	22,943 (88)	10,035 (87)	4241 (87)	977 (86)	
Yes	5875 (12)	438 (13)	3122 (12)	1490 (13)	662 (13)	163 (14)	0.002^d^
If yes							
One	1642 (28)	127 (29)	906 (29)	390 (26)	175 (26)	44 (27)	
Both	4233 (72)	311 (71)	2216 (71)	1100 (74)	487 (74)	119 (73)	0.270^d^
Statin use							
No	40,279 (86)	2902 (85)	22,655 (87)	9812 (85)	4029 (82)	881 (77)	
Yes	6763 (14)	507 (15)	3410 (13)	1713 (15)	874 (18)	259 (23)	<0.001^d^
Alcohol use							
No	12,319 (26)	979 (29)	6269 (24)	2999 (26)	1581 (32)	491 (43)	
Yes (any)	34,183 (73)	2382 (70)	19,538 (75)	8385 (73)	3246 (66)	643 (55)	<0.001^d^
Missing	540 (1)	48 (1)	258 (1)	141 (1)	77 (2)	17 (1)	
If yes, median units per week (IQR)	7 (3–12)	7 (3–12)	7 (4–12)	7 (3–12)	6 (3–12)	6 (2–10)	<0.001^b^
Any exercise							
No	8266 (18)	668 (20)	4241 (16)	2051 (18)	1004 (20)	302 (26)	
Yes/any	34,667 (74)	2437 (71)	19,641 (75)	8434 (73)	3447 (70)	708 (62)	<0.001^d^
Missing	4109 (8)	304 (9)	2183 (8)	1040 (9)	452 (9)	130 (11)	
If yes, median units per week (IQR)	5 (3–10)	5 (3–10)	5 (3–10)	5 (3–10)	5 (2–10)	5 (2–10)	<0.001^b^
Mean age at menarche (SD)	12.9 (1.62)	13.4 (1.68)	13.0 (1.59)	12.7 (1.58)	12.4 (1.63)	12.2 (1.72)	<0.001^b^
Mean age at first pregnancy (SD)	24.5 (5.1)	24.5 (5.1)	24.7 (5.1)	24.3 (5.0)	23.8 (5.0)	23.3 (5.3)	<0.001^b^
Parity							
No	5954 (13)	511 (15)	3139 (12)	1400 (12)	679 (14)	225 (20)	
Yes	41,088 (87)	2898 (85)	22926 (88)	10,125 (88)	4224 (86)	915 (80)	<0.001^d^
Median no. of children (IQR) (if parous)	2 (2–3)	2 (2–3)	2 (2–3)	2 (2–3)	2 (2–3)	2 (2–3)	0.400^b^

*BMI* body mass index, *SD* standard deviation, *IQR* inter-quartile range, *HRT* hormonal replacement therapy.

^a^Cochrane–Armitage test for trends (2 × *n*) of Caucasian versus others.

^b^Cuzick’ test for trends.

^c^Cochrane–Armitage test for trends (2 × *n*) of post-menopausal women versus others.

^d^Cochrane–Armitage test for trends (2 × *n*).

### Associations with new total breast cancer

With a median follow-up of 5.6 [inter-quartile range (IQR): 4.7–6.4] years, there were 1142 new diagnoses of breast cancer [absolute risk: 4.6 (95% CI: 4.3–4.8) per 1000 person years] (Table 2). Of these, there were 383 screen-detected prevalent and 759 incident cancers. Of the women with cancers, the histology was DCIS in 190 (17%). There were no significant associations between median BMI at cohort entry and T-stage, and N-stage (Supplementary Table S5).

**Table 2:** Hazard ratios^a^ and 95% CIs for all new breast cancers by BMI, height and weight changes in 47,042 women in PROCAS (2009–2015), in three different models.

	*n*	Cancers	Model A	Model B	Model C
			HR (95% CIs)	HR (95% CIs)	HR (95% CIs)
Total cohort	47,042	1142			
Absolute risk (per 1000 person years)	4.567 (4.310, 4.840)				
BMI at age 20 years (kg/m^2^)					
<18.5	3409	111	1.329 (1.086, 1.625)	1.218 (0.971, 1.530)	
18.5–22.4	26,065	646	1.000	1.000	
22.5–24.9	11,525	267	0.931 (0.807, 1.074)	0.962 (0.825, 1.122)	
25.0–29.9	4903	105	0.870 (0.708, 1.069)	0.871 (0.691, 1.067)	
≥30	1140	13	0.481 (0.277, 0.830)	0.394 (0.196, 0.793)	
Per SD (3.23 kg/m^2^)			0.867 (0.812, 0.927) *P* < 0.001	0.880 (0.817, 0.948) *P* = 0.001	
Height (m) at cohort entry					
Q1 (1.20–1.54)	5229	105	1.000	1.000	
Q2 (1.55–1.59)	11,496	249	1.099 (0.875, 1.381)	1.094 (0,856, 1.398)	
Q3 (1.60–1.62)	6107	165	1.383 (1.083, 1.767)	1.432 (1.102, 1.860)	
Q4 (1.63–1.67	13030	302	1.200 (1.083, 1.767)	1.193 (0.938, 1.517)	
Q5 (≥ 1.68)	11,180	321	1.534 (1.230, 1.914	1.439 (1.129, 1.834)	
Per SD (6.5 cm)			1.137 (1.073, 1.205) *P* < 0.001	1.102 (1.033, 1.175) *P* = 0.003	
Body mass index at cohort entry (kg/m^2^)					
<18.5	365	5	0.712 (0.292, 1.737)	0.949 (0.387, 2.327)	0.880 (0.360, 2.153)
18.5–22.4	7386	140	1.000	1.000	1.000
22.5–24.9	10,535	256	1.258 (1.024, 1.546)	1.287 (1.022, 1.620)	1.353 (1.073, 1.998)
25.0–29.9	16,614	426	1.314 (1.085, 1.591)	1.432 (1.156, 1.774)	1.607 (1.292, 1.998)
≥30	12,142	315	1.355 (1.110, 1.653)	1.583 (1.263, 1.985)	2.013 (1.583, 2.558)
Per SD (5.4 kg/m^2^)			1.053 (0.993, 1.115) *P* = 0.082	1.094 (1.026, 1.167) *P* = 0.006	1.207 (1.123, 1.298) P < 0.001
Weight change (absolute—kg)^b^					
Loss ≥5 kg	1527	25	0.908 (0.595, 1.385)	0.840 (0.506, 1.395)	0.973 (0.581, 1.629)
Stable (within 5 kg)	8658	157	1.000	1.000	1.000
Gain (5–9.9 kg)	10,029	217	1.191 (0.970, 1.463)	1.237 (0.987, 1.551)	1.223 (0.976, 1.534)
Gain (10–19.9 kg)	15,309	400	1.429 (1.188, 1.719)	1.500 (1.223, 1.840)	1.480 (1.206, 1.815)
Gain (≥20 kg)	11,519	343	1.641 (1.359, 1.982)	1.768 (1.431, 2.183)	1.755 (1.421, 2.167)
Per SD (12.2 kg)			1.162 (1.101, 1.228) *P* < 0.001	1.185 (1.115, 1.260) *P* < 0.001	1.178 (1.107, 1.254) P < 0.001
Weight change (relative—%)^b^					
Loss ≥5%	2241	40	1.060 (0.722, 1.554)	0.959 (0.616, 1.495)	1.032 (0.659, 1.616)
Stable (within 5 %)	4527	76	1.000	1.000	1.000
Gain (5–14.9%)	10,389	209	1.203 (0.926, 1.565)	1.146 (0.861, 1.525)	1.136 (0.854, 1.512)
Gain (15–29.9%)	14,300	347	1.434 (1.119, 1.838)	1.460 (1.116, 1.909)	1.440 (1.101, 1.884)
Gain (≥30%)	15,583	470	1.786 (1.394, 2.275)	1.831 (1.406, 2.384)	1.778 (1.363, 2.319)
Per SD (21.3%)			1.167 (1.106, 1.231) *P* < 0.001	1.196 (1.127, 1.270) *P* < 0.001	1.177 (1.107, 1.252) *P* < 0.001
Weight change (rate: kg/year)					
Loss ≥0.5 kg/year	865	15	0.939 (0.585, 1.870)	1.045 (0.585, 1.870)	1.326 (0.730, 2.409)
Normal variation (within 0.5 kg/ year)	14,470	297	1.000	1.000	1.000
Gain (0.5–1.0 kg/year)	18,591	471	1.254 (1.084, 1.450)	1.311 (1.117, 1.537)	1.287 (1.098, 1.510)
Gain (1.0–2.0 kg/year)	10,805	298	1.443 (1.227, 1.697)	1.522 (1.271, 1.821)	1.502 (1.255, 1.799)
Gain (≥2.0 kg/year)	2311	61	1.524 (1.151, 2017)	1.582 (1.152, 2.171)	1.602 (1.167, 2.198)
Per SD (0.9 kg/year)			1.163 (1.098, 1.231) *P* < 0.001	1.184 (1.110, 1.262) *P* < 0.001	1.178 (1.104, 1.258) *P* < 0.001

*BMI* body mass index, *CI* confidence interval.

^a^Cox model with attained age as timescale.

^b^Weight changes (absolute and relative) are across the time from recall at age 20 years to cohort entry.

Model A: adjusted for age only.

Model B: adjusted for age at study entry, height, age at menarche, race (White, Asian, Black, Jewish, Others, missing), hormonal replacement therapy (ever/never user), statin (ever/never user), alcohol use (yes/no/missing), any exercise (yes/no/missing), number of children, age at first pregnancy, hysterectomy, number of ovaries removed, menopausal status (post-menopausal versus pre- and peri-menopausal).

Model C: as for model B, plus adjustment for BMI at age 20 years.

In the fully adjusted model (model B), BMI aged 20 years was inversely associated with subsequent occurrence of breast cancer [HR per SD: 0.880 (95% CI: 0.817–0.948)]. By contrast, the following were positively associated with breast cancer: height [HR per SD: 1.102 (95% CI: 1.033–1.175)]; BMI at cohort entry [HR per SD: 1.094 (95% CI: 1.026–1.167)]; absolute weight gain from age 20 years to cohort entry [HR per SD:1.185 (95% CI: 1.115–1.260)]; relative weight gain [HR per SD: 1.196 (95% CI: 1.127–1.270)]; and rate of weight gain [HR per SD: 1.184 (95% CI: 1.110–1.262)].

The addition of BMI at age 20 years to the adjusted models increased the association with breast cancer for BMI at cohort entry [HR per SD: 1.207 (95% CIs: 1.123–1.298)], but attenuated associations from absolute weight gain, relative weight gain, and rate of weight gain.

### Associations with breast cancers in post- and pre-menopausal

Of the 1142 breast cancers, there were 829 in women who were post-menopausal at cohort entry [absolute risk: 4.7 (95% CI: 4.3–5.0) per 1000 person years] and 313 in women classified as either pre- or peri-menopausal at cohort entry [absolute risk: 4.3 (95% CI:3.9–4.8) per 1000 person years] (Table 3).

**Table 3:** Hazard ratios^a^ and 95% CIs for new breast cancers by BMI, height and weight changes in post- and pre-/peri-menopausal women in PROCAS (2009–2015).

	Post-menopausal			Pre-/peri-menopausal			Test for interaction
	Model B			Model B			
	*n*	Cancers	HR (95% CIs)	*n*	Cancers	HR (95% CIs)	
Total cohort	32,278	829		14,764	313		
Absolute risk (per 1000 person years)	4.667 (4.310, 4.996)			4.323 (3.869, 4.829)			
BMI at age 20 years (kg/m^2^)							
<18.5	2349	81	1.166 (0.891, 1.525)	1060	30	1.358 (0.878, 2.070)	
18.5–22.4	17,861	478	1.000	8204	168	1.000	
22.5–24.9	8039	188	0.874 (0.727, 1.050)	3486	79	1.237 (0.932, 1.641)	
25.0–29.9	3326	71	0.797 (0.602, 1.056)	1577	34	1.082 (0.719, 1.629)	
≥30	703	11	0.505 (0.239, 1.068)	437	2	0.156 (0.022, 1.118)	
Per SD (3.23 kg/m^2^)			0.866 (0.794, 0.945) *P* = 0.001			0.919 (0.800, 1.056) *P* = 0.234	*P* = 0.354
Height (m) at cohort entry							
Q1 (1.20–1.54)	3955	85	1.000	1274	20	1.000	
Q2 (1.55–1.59)	8232	196	1.087 (0.826, 1.429)	3264	53	1.124 (0.656, 2,352)	
Q3 (1.60–1.62)	4291	122	1.379 (1.026, 1.853)	1816	43	1.896 (0.884, 4.067)	
Q4 (1.63–1.67	8737	207	1.078 (0.836, 1.442)	4293	95	1.870 (0.783, 4.464)	
Q5 (≥1.68)	7063	219	1.357 (1.029, 1.788)	4117	102	2.383 (0.727, 7.806)	
Per SD (6.5 cm)			1.080 (1.002, 1.165) *P* = 0.044			1.154 (1.023, 1.301) *P* = 0.019	*P* = 0.484
	Model C			Model C			
	*n*	Cancers	HR (95% CIs)	*n*	Cancers	HR (95% CIs)	
Body mass index at cohort entry (kg/m^2^)							
<18.5	266	4	1.030 (0.374, 2.829)	99	1	0.610 (0.084, 4.440)	
18.5–22.4	4820	90	1.000	2566	50	1.000	
22.5–24.9	7087	185	1.560 (1.166, 2.086)	3448	71	1.031 (0.700, 1.521)	
25.0–29.9	11,711	322	1.882 (1.431, 2.476)	4903	104	1.158 (0.797, 1.680)	
≥30	8394	228	2.382 (1.770, 3.204)	3748	87	1.410 (0.921, 2.156)	
Per SD (5.4 kg/m^2^)			1.265 (1.164, 1.374) *P* < 0.001			1.053 (0.903, 1.228) *P* = 0.512	*P* = 0.146
Weight change (absolute—kg)^b^							
Loss ≥5 kg	1075	19	1.040 (0.561, 1.927)	452	6	0.878 (0.340, 2.267)	
Stable (within 5 kg)	5783	104	1.000	2875	53	1.000	
Gain (5–9.9 kg)	6795	161	1.408 (1.068, 1.857)	3234	56	0.902 (0.604, 1.348)	
Gain (10–19.9 kg)	10,500	285	1.623 (1.259, 2.091)	4809	115	1.227 (0.866, 1.739)	
Gain (≥20 kg)	8125	260	2.013 (1.554, 2.607)	3394	83	1.280 (0.877, 1.866)	
Per SD (12.2 kg)			1.227 (1.142, 1.319) *P* < 0.001			1.046 (0.922, 1.185) *P* = 0.486	*P* = 0.036
Weight change (relative—%)^b^							
Loss ≥5%	1609	30	1.109 (0.645, 1.905)	632	10	0.955 (0.424, 2.151)	
Stable (within 5%)	3005	49	1.000	1522	27	1.000	
Gain (5–14.9%)	6871	145	1.261 (0.880, 1.806)	3518	64	0.937 (0.583, 1.504)	
Gain (15–29.9%)	9710	247	1.623 (1.157, 2.879)	4590	100	1.140 (0.730, 1.781)	
Gain (≥30%)	11,083	358	2.063 (1.478, 2.879)	4502	112	1.291 (0.825, 2.021)	
Per SD (21.3%)			1.227 (1.142, 1.317) *P* < 0.001			1.041 (0.919, 1.181) *P* = 0.528	*P* = 0.027
Weight gain (rate: kg/year)							
Loss ≥0.5 kg/year	520	10	1.307 (0.603, 2.837)	345	5	1.247 (0.483, 3.222)	
Normal variation (within 0.5 kg/year)	10,616	223	1.000	3854	74	1.000	
Gain (0.5–1.0 kg/year)	13,085	359	1.372 (1.140, 1.651)	5506	112	1.063 (0.775, 1.457)	
Gain (1.0–2.0 kg/year)	6916	202	1.552 (1.251, 1.924)	3889	96	1.351 (0.972, 1.878)	
Gain (≥2.0 kg/year)	1141	35	2.053 (1.385, 3.041)	1170	26	1.047 (0.613, 1.787)	
Per SD (0.34 kg/year)			1.234 (1.145, 1.329) *P* < 0.001			1.079 (0.952, 1.223) *P* = 0.235	*P* = 0.012

*BMI* body mass index, *CI* confidence interval.

^a^Cox model with attained age as timescale.

^b^Weight changes (absolute and relative) are across the time from recall at age 20 years to cohort entry.

Model B: adjusted for age at study entry, height, age at menarche, race (White, Asian, Black, Jewish, Others, missing), hormonal replacement therapy (ever/never user), statin (ever/never user), alcohol use (yes/no/missing), any exercise (yes/no/missing), number of children, age at first pregnancy, hysterectomy, and number of ovaries removed.

Model C: as for model B, plus adjustment for BMI at age 20 years.

Among post-menopausal women, similar to the findings for all breast cancers, BMI aged 20 years was inversely associated with subsequent occurrence of breast cancer [HR per SD:0.866 (95% CIs: 0.794–0.945). Similar again, the following were positively associated with breast cancer: height [HR per SD:1.080 (95% CIs: 1.002–1.165)]; BMI at cohort entry [HR per SD: 1.265 (95% CIs: 1.164–1.374)]; absolute weight gain [HR per SD: 1.227 (95% CIs: 1.142–1.319)]; relative weight gain [HR per SD: 1.227 (95% CIs: 1.142–1.317)]; and rate of weight gain [HR per SD: 1.234 (95% CIs: 1.145–1.329)].

By contrast, among pre- and peri-menopausal women, there was a positive association with height [HR per SD: 1.154 (95% CIs: 1.023–1.301)], but no associations with subsequent occurrence of breast cancer associated with BMI at age 20 years, absolute weight gain, relative weight gain, and rate of weight gain.

Given the difference in risk association patterns by menopausal status, we tested for interactions. This suggested modest effect modification by menopausal status for absolute weight gain (*P*_interaction_ = 0.036), relative weight gain (*P*_interaction_ = 0.027), and rate of weight gain (*P*_interaction_ = 0.012), but not for BMI at cohort entry.

### Stratification by BMI aged 20 years

We explored whether BMI aged 20 years modified the relationship between subsequent weight changes to BMI at cohort entry and breast cancers, stratified at the 75th percentile of BMI aged 20 years (23.4 kg/m^2^).

A BMI ≥23.4 kg/m^2^ aged 20 years was strongly predictive for being either overweight or obese at cohort entry [odds ratio: 5.687 (95% CIs: 5.415–5.974) (Supplementary Table S6). Seventy-six per cent of women with a BMI <23.4 kg/m^2^ aged 20 years gained weight (>5 kg) compared with only 24% of women with a BMI ≥23.4 kg/m^2^ aged 20 years (*P* < 0.001). Patterns were similar whether women were post- or pre-/peri-menopausal at cohort entry Supplementary (Table S6). Of the 829 breast cancers occurring in post-menopausal women, the absolute risk per 1000 person years was 4.9 (95% CI: 4.5–5.3) among women with a BMI aged 20 years <23.4 kg/m^2^, and that it was 4.0 (95% CI: 3.4–4.6) among women with a BMI aged 20 years ≥23.4 kg/m^2^ (Table 4).

**Table 4:** Hazard ratios^a^ and 95% CIs for post-menopausal breast cancers by current BMI and weight changes according to BMI category at age 20 years in PROCAS (2009–2015).

	BMI at age 20 years <23.4 kg/m^2 b^			BMI at age 20 years ≥23.4 kg/m^2 b^			Test for interaction
	Model B			Model B			
	*n*	Cancers	HR (95% CIs)	*n*	Cancers	HR (95% CIs)	
Total cohort	24,121	652		8157	177		
Absolute risk (per 1000 person years)	4.904 (4.542, 5.295)			3.962 (3.418, 4.590)			
Body mass index at cohort entry (kg/m^2^)							
<18.5	256	3	0.904 (0.284, 2.883)	10	1	5.340 (0.583, 48.93)	
18.5–22.4	4496	86	1.000	324	4	1.000	
22.5–24.9	6262	174	1.543 (1.144, 2.081)	725	11	0.905 (0.272, 3.011)	
25.0–29.9	8910	250	1.692 (1.273, 2.250)	2801	72	1.648 (0.597, 4.546)	
≥30	4097	139	2.346 (1.723, 3.194)	4297	89	1.346 (0.489, 3.703)	
Per SD (5.4 kg/m^2^)			1.301 (1.183, 1.430) *P* < 0.001			0.984 (0.843, 1.148) *P* = 0.836	*P* = 0.005
Weight change (absolute—kg)							
Loss ≥5 kg	320	4	0.494 (0.121, 2.020)	755	15	1.007 (0.467, 2.171)	
Stable (within 5 kg)	4419	80	1.000	1364	24	1.000	
Gain (5–9.9 kg)	5351	125	1.356 (0.987, 1.862)	1444	36	1.698 (0.967, 2.980)	
Gain (10–19.9 kg)	8131	233	1.747 (1.310, 2.330)	2369	52	1.292 (0.753, 2.221)	
Gain (≥20 kg)	5900	210	2.300 (1.715, 3.084)	2225	50	1.315 (0.758, 2.283)	
Per SD (12.2 kg)			1.312 (1.210, 1.424) *P* < 0.001			1.054 (0.919, 1.209) *P* = 0.451	*P* = 0.014
Weight change (relative—%)							
Loss ≥5%	674	10	0.826 (0.362, 1.881)	935	20	1.240 (0.543, 2.832)	
Stable (within 5%)	2181	37	1.000	824	12	1.000	
Gain (5–14.9%)	5203	104	1.089 (0.715, 1.660)	1668	41	1.900 (0.945, 3.819)	
Gain (15–29.9%)	7317	194	1.672 (1.133, 2.467)	2393	53	1.522 (0.765, 3.028)	
Gain (≥30%)	8746	307	2.267 (1.550, 3.317)	2337	51	1.506 (0.751, 3.019)	
Per SD (21.3%)			1.288 (1.195, 1.390) *P* < 0.001			1.058 (0.899, 1.246) *P* = 0.494	*P* = 0.050
Weight gain (rate: kg/year)							
Loss ≥0.5 kg/year	86	1	Not estimable	434	9	0.892 (0.401, 1.985)	
Normal variation (within 0.5 kg/year)	8000	166	1.000	2616	57	1.000	
Gain (0.5–1.0 kg/year)	10,191	292	1.541 (1.247, 1.904)	2894	67	0.983 (0.668, 1.449)	
Gain (1.0–2.0 kg/year)	5100	165	1.861 (1.458, 2.375)	1816	37	0.880 (0.551, 1.407)	
Gain (≥2.0 kg/year)	744	28	2.762 (1.778, 4.291)	397	7	0.800 (0.333, 1.924)	
Per SD (0.34 kg/year)			1.328 (1.220, 1.445) *P* < 0.001			1.049 (0.914, 1.204) *P* = 0.497	*P* = 0.028

*BMI* body mass index, *CI* confidence interval.

Model B: adjusted for age at study entry, age at menarche, race (White, Asian, Black, Jewish, Others, missing), hormonal replacement therapy (ever/never user), statin (ever/never user), alcohol use (yes/no/missing), any exercise (yes/no/missing), number of children, age at first pregnancy, hysterectomy, and number of ovaries removed.

^a^Cox model with attained age as timescale.

^b^Cut-off of 23.4 kg/m^2^ is 75th centile; clinically plausible cut-off to define ‘overweight’ in aged 20 years.

This led us to test whether this stratification modified the relationships with subsequent occurrence of breast cancer according to weight change. Among women who were post-menopausal at study entry and who had a recall BMI at aged 20 years <23.4 kg/m^2^, the following were positively associated with subsequent occurrence of breast cancer: BMI at cohort entry [HR per SD: 1.301 (95% CIs: 1.183–1.430)]; absolute weight gain [HR per SD: 1.312 (95% CIs: 1.210–1.424)]; relative weight gain [HR per SD: 1.288 (95% CIs: 1.195–1.390)]; and rate of weight gain [HR per SD: 1.328 (95% CIs: 1.220–1.445)].

We tested for interactions. This suggested an effect modification by strata of recall BMI aged 20 years for absolute weight gain (*P*_interaction_ = 0.014), relative weight gain (*P*_interaction_ = 0.050) and rate of weight gain (*P*_interaction_ = 0.028), and BMI at cohort entry (*P*_interaction_ = 0.005).

### Sensitivity analyses

We repeated the main models excluding DCIS cases (Supplementary Table S7) and prevalent cancers (Supplementary Table S8) and found essentially identical results. We explored adjusting models of associations of BMI aged 20 years with breast cancer by BMI at cohort entry and found stronger inverse associations (Supplementary Table S9). Because of the correlation between BMI aged 20 years and BMI at cohort entry (Pearson’s correlation = 0.51), we were concerned that these might have been over-fitted estimates. Finally, we tested and found no evidence of survivor bias (Table S10).

## Discussion

### Main findings

In a large UK mainly urban (mainly Caucasian) population, we found the following. First, we confirmed that young adulthood BMI is inversely associated with breast cancer risk, while adult weight gain is positively associated with breast cancer among women who were post-menopausal at cohort entry. Second, standardised estimates for adult weight gain expressed as absolute, relative or as a rate, were broadly similar. Third, the selection of BMI aged 20 years cut-off of 23.4 kg/m^2^ (75th percentile of BMI distribution at age 20 years) predicted for subsequent weight gainers and was an effect modifier for the weight gain/breast cancer risk association. The novel finding was that adult weight gain increased post-menopausal breast cancer risk among women who had a BMI <23.4 kg/m^2^ aged 20 years, but not among women who had a BMI >23.4 kg/m^2^ aged 20 years.

### Context of other studies

Previous reviews^3,5^ and the recent Pre-menopausal Breast Cancer Collaborative Group^27^ analysis indicate that young adulthood BMI is inversely associated with risk of pre-menopausal as well as post-menopausal breast cancer. Our analysis did not show the former, reflecting the small numbers of pre- and peri-menopausal cancers in our cohort

For adult weight gain, our study found positive associations between weight gain and breast cancer in post-menopausal but not pre- and peri-menopausal women. This concurs with the Keum et al.^6^ meta-analysis and the WCRF 2018 report.^5^ The summary estimates from the latter were: post-menopausal breast cancer risk (15 studies; summary estimate per 5 kg: 1.06, 95% CI: 1.05–1.08); pre-menopausal breast cancer risk (5 studies; summary estimate per 5 kg: 0.99, 95% CI: 0.96–1.03).

We tested for interactions between BMI aged 20 years, weight changes and breast cancer risk. We found that the selection of BMI aged 20 years cut-off of 23.4 kg/m^2^ was an effect modifier for determining subsequent breast cancer risk in women who were post-menopausal at cohort entry. The only other study to perform a similar stratification was reported by Eliassen et al.^12^ for the Nurses’ Health Study cohort. Using a cut-off of 21 kg/m^2^, they found stronger associations between weight gain (expressed only as absolute weight change) and breast cancer in women with BMI at age 18 years <21 kg/m^2^ compared with those with BMI values >21 kg/m^2^. This effect modification was significant (*P*_interaction_ = 0.04). Their analysis was limited to post-menopausal women.

In the interpretation of the similar findings from our study and those from Eliassen et al.,^12^ it is important to note that these cut-points are population specific. As adolescent obesity trends evolve with time, these cut-points are likely to differ (most likely upwards) in future studies.

### Limitations and strengths

There are potential study limitations. First, the height and weight were self-reported, which may bias BMI–cancer associations. This bias tends to underestimate weight in older individuals and in heavier individuals.^28^ The equivalent arguments might apply to recall BMIs at age 18–21 years. However, a recent meta-analysis indicates that there is little bias for these recall estimates.^29^ We tested BMI distributions of our study against a UK population data contemporaneous with our population for the respective age strata and found similar BMI parameters. Second, our findings on associations with BMI aged 20 years may not be generalisable to today’s populations, as BMI distributions for equivalent age groups have shifted upwards.^30^ Third, the majority of the studied population were Caucasian and thus results might not be generalisable to other ethnic groups. The risk of breast cancer varies across racial groups; and relationships between high BMI and breast cancer risk and the changes in anthropometric measures vary by racial groups.^31^ Fourth, we included prevalent cancers in our main analysis and there might be confounding on weight change due to the presence of disease (i.e. reverse causality). This confounding is likely to be exceptionally small in this asymptomatic screen-detected population, and the exclusion of prevalent cancers in our sensitivity analysis made no material impact on risk estimates. Fifth, there is risk of residual confounding from measured and unmeasured factors. Even among the measured confounders, the classifications of confounders like alcohol consumption and exercise were relatively crude. Finally, the increased risk of weight gain and the development of post-menopausal breast cancer are generally limited to non-HRT users.^5,6^ We did not stratify our analyses in post-menopausal breast cancer by HRT status because of diminishing sample sizes.

Strengths of the present study include (first) its prospective design and a contemporaneous population. Second, there are concerns in the literature that obesity (and its associated co-morbidities)^32^ is associated with reduced uptake for breast cancer screening, hence introducing an ascertainment bias. This has not been found in UK populations.^33^ Third, even with representative BMI distribution in the screened population, a further bias might occur if excess adiposity influenced cancer stage detection on mammography. We found this not to be the case. Fourth, we addressed changes in weight across adulthood using three parameters and standardised the derived risk estimates. We found risks to be similar for all three parameterisations. Finally, we undertook sensitivity analyses and found our models to be consistent.

### Clinical implications

Our results support the existing literature that weight gain more strongly associates with breast cancer risk than a ‘once-only’ measure of BMI and an emerging theme that cancer prevention should be targeted across the life course.^34^ Thus, in future studies, adult weight gain might replace BMI as a risk factor in breast cancer risk prediction models. Furthermore, weight gain is more easily interpreted by providers and policy makers, and better understood in weight management programmes. The biological mechanisms underlying the paradoxical ‘protective’ association between elevated BMI in young adulthood (relative to women with BMI aged 20 years <23.4 kg/m^2^) and post-menopausal breast cancer are unclear, but there are a number of potential pathways. Baer et al.^35^ and Schoemaker et al.^27^ summarised some of these as follows. First, elevated BMI in adolescent girls is associated with higher basal insulin levels and decreased plasma levels of sex hormone binding globulin, leading to increased free oestrogen and testosterone. Second, high levels of androgens in adolescent obese girls are associated with metabolic features similar to ‘polycystic ovary syndrome, greater frequency of anovulatory cycles, and reduced fertility later in life. Third, Hilakivi-Clarke et al.^36^ postulated ‘that early oestrogen exposure may reduce breast cancer risk by increasing the expression of tumour suppressor genes such as *BRCA1*’ resulting in a more stable mammary gland epithelial genome. Fourth, Dowsett and Folkerd^37^ point out (contrary to common perception) that in the young woman, serum oestrogen levels are not elevated in obesity as this is regulated through a negative hypothalamic feedback. However, progesterone levels are reduced and this better explains the apparent reduced risk of breast cancer in obese pre-menopausal women. Further mechanisms may include altered circulating insulin growth factor-1 (IGF-1) levels in adulthood (women who are obese at age 18 years have lower mean IGF-1 levels compared with low normal weight women)^38^ or mediation through lower adult mammographic density.^39^ Using mediation analysis, Rice et al.^4^ estimated that, in pre-menopausal women, the associations between adolescent somatotype and breast cancer risk was substantially mediated by per cent mammographic density (per cent mediated = 73%), but in post-menopausal women, the proportion of the associations with adolescent somatotype that was mediated by per cent mammographic density was less (per cent mediated = 26%).

### Unanswered questions and future research

Important questions remain regarding how to best quantify the cumulative effects of excess body weight over several decades, the differential effect of key weight change periods during the life course, and interactions with other risk factors. This knowledge is equally required to inform prediction models and risk stratification approaches.^40^ Ideally the effects of weight change over time on breast cancer risk would be assessed in longitudinal studies with repeated measurements commencing in early adulthood. The statistical handling of these data will be complex and computationally demanding. Finally, it remains to be seen whether preventing weight gain and/or reducing BMI via effective interventions in adult populations reduces breast cancer risk and forms the basis for public health strategies to prevent breast cancer worldwide.

## Supplementary information

Supplemental material

## Data Availability

The datasets supporting the conclusions of this article are stored in a secured research database and may be available upon presentation of formal approval.
