# Supplementary material for: Young adulthood body mass index, adult weight gain and breast cancer risk: the PROCAS Study (United Kingdom)
Source: Br J Cancer. 2020 Mar 23;122(10):1552–61. doi: 10.1038/s41416-020-0807-9 (PMC7217761; doi:10.1038/s41416-020-0807-9)
Supplement: Supplementary file 1 — Supplemental material [file 41416_2020_807_MOESM1_ESM.docx]

Supplemental material

**Young adulthood body mass index, adult weight gain and breast cancer risk: the PROCAS Study (United Kingdom)**

Andrew G Renehan et al.

**Table S1 Summary of the literature of studies evaluating weight change and subsequent breast cancer risk, and whether there was adjustment for young adulthood BMI or weight**

From two meta-analyses (2015^1^ and 2018^2^), we included studies that reported young adulthood BMI or weight, weight change and breast cancer risk. And updated to include studies after search dates in the above systematic reviews.

|  | **Authors** | **Year** | **Cohort** | **No. Breast cancers** | **Pre-meno** | **Post_meno** | **Any adjustment for BMI age 18-21** | **Estimates with and without BMI age 18_21** | **Stratification by BMI age 18-21** | **Comments** |
| --- | --- | --- | --- | --- | --- | --- | --- | --- | --- | --- |
| 1 | Feigelson et al.^3^ | 2004 | CPS II | 1934 | 0 | 1 | 0 | 0 | 0 |  |
| 2 | Lahmann et al.^4^ | 2005 | EPIC | 1358 | 1 | 1 | 1 | 0 | 0 |  |
| 3 | Eliasson et al.^5^ | 2006 | Nurses' Health Study | 4393 | 0 | 1 | 1 | 0 | 1 | Cut-off at BMI at age 18 years = 21 kg/m^2^ |
| 4 | Palmer et al.^6^ | 2007 | Black Women's Health Study | 1062 | 1 | 1 | 1 | 1 | 0 | No major change in risk estimates after adjustment for BMI at age 21 years |
| 5 | Ahn et al.^7^ | 2007 | AARP | 2111 | 0 | 1 | 1 | 0 | 0 |  |
| 6 | Kawai et al.^8^ | 2010 | Miyagi Cohort | 108 | 0 | 1 | 1 | 0 | 0 |  |
| 7 | Alsakar et al.^9^ | 2013 | HUNT Study | 900 | 0 | 1 | 1 | 0 | 0 |  |
| 8 | Catsburg et al.^10^ | 2014 | Canadian Study of Diet, Lifestyle and Health | 1097 | 1 | 1 | 0 | 0 | 0 |  |
| 9 | Rosner et al.^11^ | 2017 | Nurses' Health Study | 4965 | 1 | 1 | 1 | 1 | 0 | Adjustment for somatotype at age 10 years |
|  | Present study |  | PROCAS | 1142 | 1 | 1 | 1 | 1 | 1 |  |

**Table S2 Recall median (IQR) BMI values at age 20 years according to decade of 20^th^ birthday, in 47042 women in PROCAS (2009-2015)**

| **PROCAS cohort** | | | **Literature** | |
| --- | --- | --- | --- | --- |
| Decade of 20^th^ birthday | Mean (SD) | Median (IQR) | Decade of 20^th^ birthday | Median (IQR) |
| 1950 to 1959 | 22.0 (2.7) | 21.9  (20.2-23.4) | No data | No data |
| 1960 to 1969 | 22.0 (3.0) | 21.7  (20.1-23.4) | No data | No data |
| 1970 to 1979 | 22.1 (3.3) | 21.7  (20.0-23.4) | No data | No data |
| 1980 to 1989 | 22.2 (3.5) | 21.7  (20.0-23.6) | 1980 to 1989^12^ | 21.8  (20.1-23.9) |

SD: standard deviation. IQR: interquartile range

**Table S3 Baseline characteristics by menopausal status illustrating that missing menopausal status is not random, PROCAS (2009-2015)**

|  | **Peri-menopausal** | **Post-menopausal** | **Pre-menopausal** | **Missing** |
| --- | --- | --- | --- | --- |
|  |  |  |  |  |
| **N** | 9042 | 33278 | 5722 | 2368 |
| **Median age (IQR)** | 52.3  (50.7-54.9) | 61.7  (56.8-66.0) | 49.9  (48.3-51.2) | 50.6  (49.3-51.2) |
| **Age categories** |  |  |  |  |
| 44.0 to 53.9 years | 6202 (69) | 4505 (14) | 5409 (95) | 2056 (87) |
| 54.0 to 59.9 years | 2402 (27) | 8622 (27) | 198 (3) | 179 (8) |
| ≥ 60 years | 438 (5) | 19151 (59) | 115 (2) | 133 (6) |
| **Median BMI kg/m^2^ (IQR)** | 26.1  (23.4-30.1) | 26.5  (23.7-30.2) | 26.0  (23.1-30.0) | 26.5  (23.4-30.8) |
| **Hysterectomy** |  |  |  |  |
| No | 7672 (85) | 22205 (69) | 5238 (92) | 2140 (90) |
| Yes | 1288 (14) | 9887 (31) | 446 (8) | 198 (8) |
| **Oophorectomy** |  |  |  |  |
| No | 8713 (96) | 26843 (83) | 5611 (98) | 2315 (98) |
| Yes | 329 (4) | 5435 (17) | 111 (2) | 53 (2) |
| **If yes,** |  |  |  |  |
| One | 328 (100) | 1203 (22) | 111 (100) | 53 (100) |
| Both | 1 | 4232 (78) | 0 | 0 |

BMI: body mass index. IQR: inter-quartile range.

**Table S4 Pearson correlations for anthropometric parameters in in 47042 women in PROCAS (2009-2015)**

|  | **BMI at cohort entry** | **BMI at age 20 years** | **Wt-change absolute** | **Wt-change relative** | **Wt-change rate** | **Age** |
| --- | --- | --- | --- | --- | --- | --- |
| **BMI at cohort entry** | 1.000 |  |  |  |  |  |
| **BMI at age 20 years** | 0.5161 | 1.000 |  |  |  |  |
| **Wt-change absolute** | 0.7945 | -0.1000 | 1.000 |  |  |  |
| **Wt-change relative** | 0.7712 | -0.0856 | 0.9667 | 1.000 |  |  |
| **Wt-change rate** | 0.7161 | -0.2084 | 0.9723 | 0.9310 | 1.000 |  |
| **Age** | 0.0002 | -0.0238 | 0.0057 | -0.1825 | 0.0248 | 1.000 |

**Table S5 Relationships between BMI and T and N stage (AJCC 7^th^ Edition) in women with new breast cancer during follow-up, PROCAS (2009-2015)**

| **T stage** | | | **N stage** | | |
| --- | --- | --- | --- | --- | --- |
|  | **N (%)** | **Median BMI (IQR)** |  | **N (%)** | **Median BMI (IQR)** |
| DCIS | 190 (20) | 26.4  (24.1-30.0) | N0 | 666 (80) | 26.6  (24.1-30.5) |
| T1 | 572 (59) | 27.0  (24.1-30.7) | N1 | 77 (9) | 27.3  (24.2-30.6) |
| T2 | 182 (19) | 26.4  (24.1-30.3) | N2 | 51 (6) | 28.9  (24.6-33.0) |
| T3 | 18 (2) | 27.2  (24.9-30.7) | N3 | 34 (4) | 27.3  (24.9-29.1) |
|  |  | P = 0.611 |  |  | P = 0.531 |

**Table S6 BMI at entry and adult weight changes according to whether BMI was less than or greater than 23.4 kg/m^2^ at age 20 years, in PROCAS (2009-2015)**

| **All women (N: 47042)** | | | |
| --- | --- | --- | --- |
|  | **BMI at study entry** | | |
|  | **Underweight**  **< 18.5 kg/m^2^** | **Normal**  **18.5 – 24.9 kg/m^2^** | **Overweight/ obese**  **≥ 25.0 kg/m^2^** |
| **BMI at age 20 years** |  |  |  |
| < 23.4 kg/m^2^  (row %)  (col %) | 353  (1)  (97) | 28943  (83)  (84) | 5720  (16)  (47) |
| ≥ 23.4 kg/m^2^  (row %)  (col %) | 12  (0)  (3) | 5592  (47)  (16) | 6422  (53)  (53) |
|  |  |  |  |
|  | **Absolute weight change** | | |
|  | **Loss**  **< 5 kg** | **Stable**  **-5 to +5 kg** | **Gain**  **>5 kg** |
| **BMI at age 20 years** |  |  |  |
| < 23.4 kg/m^2^  (row %)  (col %) | 432  (1)  (28) | 6677  (19)  (77) | 27907  (80)  (76) |
| ≥ 23.4 kg/m^2^  (row %)  (col %) | 1095  (9)  (72) | 1981  (16)  (23) | 8950  (74)  (24) |
|  |  |  |  |

| **Post-menopausal women (N: 32,278)** | | | |
| --- | --- | --- | --- |
|  | **BMI at study entry** | | |
|  | **Underweight**  **< 18.5 kg/m^2^** | **Normal**  **18.5 – 24.9 kg/m^2^** | **Overweight/ obese**  **≥ 25.0 kg/m^2^** |
| **BMI at age 20 years** |  |  |  |
| < 23.4 kg/m^2^  (row %)  (col %) | 256  (1)  (96) | 19768  (82)  (84) | 4097  (17)  (49) |
| ≥ 23.4 kg/m^2^  (row %)  (col %) | 10  (0)  (4) | 3850  (47)  (16) | 4297  (53)  (51) |
|  |  |  |  |
|  | **Absolute weight change** | | |
|  | **Loss**  **< 5 kg** | **Stable**  **-5 to +5 kg** | **Gain**  **>5 kg** |
| **BMI at age 20 years** |  |  |  |
| < 23.4 kg/m^2^  (row %)  (col %) | 320  (1)  (30) | 4419  (18)  (76) | 19382  (80)  (76) |
| ≥ 23.4 kg/m^2^  (row %)  (col %) | 755  (9)  (70) | 1364  (17)  (24) | 6038  (74)  (24) |
|  |  |  |  |

| **Pre-menopausal women (N: 14,764)** | | | |
| --- | --- | --- | --- |
|  | **BMI at study entry** | | |
|  | **Underweight**  **< 18.5 kg/m^2^** | **Normal**  **18.5 – 24.9 kg/m^2^** | **Overweight/ obese**  **≥ 25.0 kg/m^2^** |
| **BMI at age 20 years** |  |  |  |
| < 23.4 kg/m^2^  (row %)  (col %) | 97  (1)  (98) | 9175  (84)  (84) | 1623  (15)  (43) |
| ≥ 23.4 kg/m^2^  (row %)  (col %) | 2  (0)  (2) | 1742  (45)  (16) | 2125  (55)  (57) |
|  |  |  |  |
|  | **Absolute weight change** | | |
|  | **Loss**  **< 5 kg** | **Stable**  **-5 to +5 kg** | **Gain**  **>5 kg** |
| **BMI at age 20 years** |  |  |  |
| < 23.4 kg/m^2^  (row %)  (col %) | 112  (1)  (25) | 2258  (21)  (78) | 8525  (78)  (75) |
| ≥ 23.4 kg/m^2^  (row %)  (col %) | 340  (9)  (75) | 617  (16)  (21) | 2912  (75)  (25) |
|  |  |  |  |

**Table S7 Hazard ratios* and 95% CIs for new breast cancers by BMI, height and weight changes in post- and pre-/peri-menopausal women in PROCAS (2009-2015) excluding DCIS cases**

|  | **Post-menopausal** | | | **Pre-/ peri-menopausal** | | |  |
| --- | --- | --- | --- | --- | --- | --- | --- |
|  |  |  | **Model B** |  |  | **Model B** |  |
|  | **n** | **cancers** | **HR**  **(95% CIs)** | **n** | **cancers** | **HR**  **(95% CIs)** | **Test for interaction** |
|  |  |  |  |  |  |  |  |
| **Total cohort** | 32278 | 687 |  | 14764 | 265 |  |  |
|  |  |  |  |  |  |  |  |
| **Absolute risk** (per 1000 person years) | 3.868  (3.589, 4.167) | |  | 3.660  (3.244, 4.128) | |  |  |
|  |  |  |  |  |  |  |  |
| **BMI at age 20 years (kg/m^2^)** |  |  |  |  |  |  |  |
| < 18.5 | 2349 | 67 | 1.116  (0.828, 1.504) | 1060 | 26 | 1.315  (0.824, 2.099) |  |
| 18.5 to 22.4 | 17861 | 391 | 1.000 | 8204 | 142 | 1.000 |  |
| 22.5 to 24.9 | 8039 | 160 | 0.908  (0.744, 1.108) | 3486 | 69 | 1.255  (0.924, 1.703) |  |
| 25.0 to 29.9 | 3326 | 59 | 0.810  (0.596, 1.099) | 1577 | 27 | 1.085  (0.697, 1.689) |  |
| ≥ 30 | 703 | 10 | 0.607  (0.286, 1.285) | 437 | 1 | 0.177  (0.025, 1.272) |  |
| Per SD (3.23 kg/m^2^) |  |  | 0.878  (0.799, 0.964)  P = 0.007 |  |  | 0.942  (0.814, 1.090)  P = 0.424 | P = 0.306 |
|  |  |  |  |  |  |  |  |
| **Height (m) at study entry** |  |  |  |  |  |  |  |
| Q1 (1.20-1.54) | 3955 | 73 | 1.000 | 1274 | 14 | 1.000 |  |
| Q2 (1.55-1.59) | 8232 | 157 | 1.056  (0.784, 1.424) | 3264 | 43 | 1.317  (0.688, 2.519) |  |
| Q3 (1.60-1.62) | 4291 | 96 | 1.298  (0.939, 1.796) | 1816 | 37 | 1.995  (1.028, 3.870) |  |
| Q4 (1.63-1.67 | 8737 | 172 | 1.083  (0.806, 1.456) | 4293 | 82 | 1.924  (1.041, 3.556) |  |
| Q5 (≥ 1.68) | 7063 | 189 | 1.401  (1.040, 1.887) | 4117 | 89 | 2.302  (1.244, 4.261) |  |
| Per SD (6.5 cm) |  |  | 1.102  (1.015, 1.196)  P = 0.020 |  |  | 1.239  (1.089, 1.409)  P = 0.001 | P = 0.231 |
|  |  |  |  |  |  |  |  |
|  |  |  | **Model C** |  |  | **Model C** |  |
|  | **n** | **cancers** | **HR**  **(95% CIs)** | **n** | **cancers** | **HR**  **(95% CIs)** |  |
|  |  |  |  |  |  |  |  |
| **Body mass index at study entry (kg/m^2^)** |  |  |  |  |  |  |  |
| < 18.5 | 266 | 4 | 1.225  (0.443, 3.387) | 99 | 1 | 0.668  (0.091, 4.890) |  |
| 18.5 to 22.4 | 4820 | 72 | 1.000 | 2566 | 43 | 1.000 |  |
| 22.5 to 24.9 | 7087 | 152 | 1.532  (1.113, 2.109) | 3488 | 62 | 1.017  (0.671, 1.543) |  |
| 25.0 to 29.9 | 11711 | 261 | 1.847  (1.367, 2.494) | 4903 | 88 | 1.086  (0.727, 1.622) |  |
| ≥ 30 | 8394 | 198 | 2.501  (1.811, 3.454) | 3748 | 71 | 1.257  (0.793, 1.994) |  |
| Per SD (5.4 kg/m^2^) |  |  | 1.275  (1.165, 1.395)  P < 0.001 |  |  | 1,010  (0.854, 1.195)  P = 0.905 | P = 0.101 |
|  |  |  |  |  |  |  |  |
| **Weight change (absolute – kg)** |  |  |  |  |  |  |  |
| Loss ≥ 5 kg | 1075 | 14 | 0.897  (0.443, 1.817) | 452 | 5 | 0.971  (0.371, 2.540) |  |
| Stable (within 5 kg) | 5783 | 85 | 1.000 | 2875 | 45 | 1.000 |  |
| Gain (5 to 9.9 kg) | 6795 | 126 | 1.307  (0.964, 1.772) | 3234 | 47 | 0.903  (0.585, 1.395) |  |
| Gain (10 to 19.9 kg) | 10500 | 239 | 1.602  (1.216, 2.111) | 4809 | 100 | 1.222  (0.838, 1.782) |  |
| Gain (≥ 20 kg) | 8125 | 223 | 2.044  (1.546, 2.705) | 3394 | 68 | 1.210  (0.797, 1.814) |  |
| Per SD (12.2 kg) |  |  | 1.236  (1.143, 1.336)  P < 0.001 |  |  | 1.001  (0.882, 1.157)  P = 0.887 | P = 0.018 |
|  |  |  |  |  |  |  |  |
| **Weight change (relative - %)** |  |  |  |  |  |  |  |
| Loss ≥ 5% | 1609 | 24 | 1.204  (0.663, 2.186) | 632 | 9 | 1.065  (0.465, 2.436) |  |
| Stable (within 5 %) | 3005 | 38 | 1.000 | 1522 | 24 | 1.000 |  |
| Gain (5 to 14.9%) | 6871 | 119 | 1.363  (0.910, 2.044) | 3518 | 55 | 0.931  (0.561, 1.544) |  |
| Gain (15 to 29.9%) | 9710 | 203 | 1.736  (1.184,, 2.546) | 4590 | 84 | 1.100  (0.683, 1.771) |  |
| Gain (≥ 30%) | 11083 | 303 | 2.281  (1.565, 3.323) | 4502 | 93 | 1.189  (0.735, 1.924) |  |
| Per SD (21.3%) |  |  | 1.234  (1.142, 1.333)  P < 0.001 |  |  | 1.012  (0.882, 1.161)  P = 0.861 | P = 0.014 |
|  |  |  |  |  |  |  |  |
| **Weight gain (rate: kg/year)** |  |  |  |  |  |  |  |
| Loss ≥ 0.5 kg/year | 520 | 7 | 1.330  (0.575, 3.075) | 345 | 5 | 1.333  (0.509, 3.479) |  |
| Normal variation  (within 0.5 kg/ year) | 10616 | 179 | 1.000 | 3854 | 64 | 1.000 |  |
| Gain (0.5 to 1.0 kg/year) | 13085 | 300 | 1.401  (1.142, 1.717) | 5506 | 94 | 0.984  (0.701, 1.381) |  |
| Gain (1.0 to 2.0 kg/year) | 6916 | 175 | 1.663  (1.317, 2.101) | 3889 | 82 | 1.288  (0.907, 1.830) |  |
| Gain (≥ 2.0 kg/year) | 1141 | 26 | 1.911  (1.226, 2.981) | 1170 | 20 | 0.887  (0.488, 1.613) |  |
| Per SD (0.34 kg/year) |  |  | 1.247  (1.151, 1.351)  P < 0.001 |  |  | 1.053  (0.748, 1.482)  P = 0.769 | P = 0.014 |
|  |  |  |  |  |  |  |  |

BMI: body mass index. CI: confidence interval.

*Cox model with attained age as timescale.

Model B: adjusted for age at study entry, height, age at menarche, race (White, Asian, Black, Jewish, Others, missing) hormonal replacement therapy (ever/never user), statin (ever/never user), alcohol consumption (yes/ no/ missing), exercise (yes/ no/ missing), number of children, age at first pregnancy, hysterectomy, number of ovaries removed.

Model C: as for model B, plus adjustment for BMI at age 20 years

**Table S8 Hazard ratios* and 95% CIs for new breast cancers by BMI, height and weight changes in post- and pre-/peri-menopausal women in PROCAS (2009-2015) excluding prevalent breast cancer cases**

|  | **Post-menopausal** | | | **Pre-/ peri-menopausal** | | |  |
| --- | --- | --- | --- | --- | --- | --- | --- |
|  |  |  | **Model B** |  |  | **Model B** |  |
|  | **n** | **cancers** | **HR**  **(95% CIs)** | **n** | **cancers** | **HR**  **(95% CIs)** | **Test for interaction** |
|  |  |  |  |  |  |  |  |
| **Total cohort** | 32278 | 556 |  | 14764 | 203 |  |  |
|  |  |  |  |  |  |  |  |
| **Absolute risk** (per 1000 person years) | 3.130  (2.881, 3.401) | |  | 2.804  (2.443, 3.217) | |  |  |
|  |  |  |  |  |  |  |  |
| **BMI at age 20 years (kg/m^2^)** |  |  |  |  |  |  |  |
| < 18.5 | 2349 | 50 | 1.023  (0.725, 1.445) | 1060 | 22 | 1.441  (0.868, 2.396) |  |
| 18.5 to 22.4 | 17861 | 327 | 1.000 | 8204 | 114 | 1.000 |  |
| 22.5 to 24.9 | 8039 | 126 | 0.861  (0.688, 1.078) | 3486 | 47 | 1.086  (0.757, 1.557) |  |
| 25.0 to 29.9 | 3326 | 45 | 0.762  (0.538, 1.078) | 1577 | 18 | 0.850  (0.491, 1.471) |  |
| ≥ 30 | 703 | 8 | 0.538  (0.222, 1.307) | 437 | 2 | 0.229  (0.032, 1.651) |  |
| Per SD (3.23 kg/m^2^) |  |  | 0.866  (0.779, 0.964)  P = 0.008 |  |  | 0.868  (0.725, 1.039)  P = 0.122 | P = 0.851 |
|  |  |  |  |  |  |  |  |
| **Height (m) at study entry** |  |  |  |  |  |  |  |
| Q1 (1.20-1.54) | 3955 | 49 | 1.000 | 1274 | 12 | 1.000 |  |
| Q2 (1.55-1.59) | 8232 | 131 | 1.226  (0.861, 1.745) | 3264 | 37 | 1.465  (0.700, 3.066) |  |
| Q3 (1.60-1.62) | 4291 | 81 | 1.602  (1.099, 2.335) | 1816 | 24 | 1.670  (0.880, 3.647) |  |
| Q4 (1.63-1.67 | 8737 | 143 | 1.263  (0.889, 1.793) | 4293 | 59 | 1.791  (0.880, 3.647) |  |
| Q5 (≥ 1.68) | 7063 | 152 | 1.656  (1.164, 2.354) | 4117 | 71 | 2.464  (1.216, 4.993) |  |
| Per SD (6.5 cm) |  |  | 1.128  (1.029, 1.236)  P = 0.010 |  |  | 1.257  (1.084, 1.458)  P = 0.003 | P = 0.271 |
|  |  |  |  |  |  |  |  |
|  |  |  | **Model C** |  |  | **Model C** |  |
|  | **n** | **cancers** | **HR**  **(95% CIs)** | **n** | **cancers** | **HR**  **(95% CIs)** |  |
|  |  |  |  |  |  |  |  |
| **Body mass index at study entry (kg/m^2^)** |  |  |  |  |  |  |  |
| < 18.5 | 266 | 4 | 1.818  (0.648, 5.103) | 99 | 1 | 0.886  (0.119, 6.577) |  |
| 18.5 to 22.4 | 4820 | 53 | 1.000 | 2566 | 30 | 1.000 |  |
| 22.5 to 24.9 | 7087 | 130 | 1.843  (1.267, 2.677) | 3488 | 50 | 1.163  (0.718, 1.882) |  |
| 25.0 to 29.9 | 11711 | 224 | 2.265 (1.589, 3.229) | 4903 | 70 | 1.239  (0.774, 1.981) |  |
| ≥ 30 | 8394 | 145 | 2.652  (1.808, 3.890) | 3748 | 52 | 1.440  (0.839, 2.471) |  |
| Per SD (5.4 kg/m^2^) |  |  | 1.278  (1.156, 1.414)  P < 0.001 |  |  | 1.086  (0.896, 1.316)  P = 0.401 | P = 0.143 |
|  |  |  |  |  |  |  |  |
| **Weight change (absolute – kg)** |  |  |  |  |  |  |  |
| Loss ≥ 5 kg | 1075 | 10 | 0.887  (0.399, 1.971) | 452 | 4 | 0.947  (0.280, 3.199) |  |
| Stable (within 5 kg) | 5783 | 70 | 1.000 | 2875 | 31 | 1.000 |  |
| Gain (5 to 9.9 kg) | 6795 | 107 | 1.323  (0.941, 1.860) | 3234 | 31 | 0.837  (0.495, 1.416) |  |
| Gain (10 to 19.9 kg) | 10500 | 195 | 1.625  (1.193, 2.212) | 4809 | 84 | 1.417  (0.912, 2.201) |  |
| Gain (≥ 20 kg) | 8125 | 174 | 1.993  (1.455, 2.730) | 3394 | 53 | 1.384  (0.856, 2.238) |  |
| Per SD (12.2 kg) |  |  | 1.237  (1.134, 1.349)  P < 0.001 |  |  | 1.080  (0.924, 1.263)  P = 0.334 | P = 0.106 |
|  |  |  |  |  |  |  |  |
| **Weight change (relative - %)** |  |  |  |  |  |  |  |
| Loss ≥ 5% | 1609 | 17 | 1.020  (0.512, 2.033) | 632 | 8 | 1.176  (0.453, 3.053) |  |
| Stable (within 5 %) | 3005 | 32 | 1.000 | 1522 | 17 | 1.000 |  |
| Gain (5 to 14.9%) | 6871 | 98 | 1.258  (0.805, 1.965) | 3518 | 34 | 0.778  (0.423, 1.428) |  |
| Gain (15 to 29.9%) | 9710 | 173 | 1.756  (1.156, 2.665) | 4590 | 71 | 1.201  (0.692, 2.086) |  |
| Gain (≥ 30%) | 11083 | 236 | 2.075  (1.373, 3.136) | 4502 | 73 | 1.219  (0.696, 2.133) |  |
| Per SD (21.3%) |  |  | 1.235  (1.132, 1.347)  P < 0.001 |  |  | 1.059  (0.908, 1.235)  P = 0.468 | P = 0.108 |
|  |  |  |  |  |  |  |  |
| **Weight gain (rate: kg/year)** |  |  |  |  |  |  |  |
| Loss ≥ 0.5 kg/year | 520 | 5 | 1.441  (0.574, 3.617) | 345 | 3 | 1.330  (0.396, 4.467) |  |
| Normal variation  (within 0.5 kg/ year) | 10616 | 145 | 1.000 | 3854 | 48 | 1.000 |  |
| Gain (0.5 to 1.0 kg/year) | 13085 | 244 | 1.455  (1.155, 1.833) | 5506 | 73 | 1.023  (0.692, 1.514) |  |
| Gain (1.0 to 2.0 kg/year) | 6916 | 139 | 1.673  (1.284, 2.179) | 3889 | 61 | 1.298  (0.860, 1.959) |  |
| Gain (≥ 2.0 kg/year) | 1141 | 23 | 2.137  (1.334, 3.451) | 1170 | 18 | 1.328  (0.715, 2.478) |  |
| Per SD (0.34 kg/year) |  |  | 1.244  (1.138, 1.361)  P < 0.001 |  |  | 1.262  (0.850, 1.876)  P = 0.249 | P = 0.055 |
|  |  |  |  |  |  |  |  |

BMI: body mass index. CI: confidence interval.

*Cox model with attained age as timescale.

Model B: adjusted for age at study entry, height, age at menarche, race (White, Asian, Black, Jewish, Others, missing) hormonal replacement therapy (ever/never user), statin (ever/never user), alcohol consumption (yes/ no/ missing), exercise (yes/ no/ missing), number of children, age at first pregnancy, hysterectomy, number of ovaries removed.

Model C: as for model B, plus adjustment for BMI at age 20 years

**Table S9 Hazard ratios* and 95% CIs for new breast cancers by BMI at age 20 years adjusted for BMI at cohort entry in post- and pre-/peri-menopausal women in PROCAS (2009-2015)**

|  | **Post-menopausal** | | | **Pre-/ peri-menopausal** | | |  |
| --- | --- | --- | --- | --- | --- | --- | --- |
|  |  |  | **Model B** |  |  | **Model B** |  |
|  | **n** | **cancers** | **HR**  **(95% CIs)** | **n** | **cancers** | **HR**  **(95% CIs)** | **Test for interaction** |
|  |  |  |  |  |  |  |  |
| **Total cohort** | 32278 | 829 |  | 14764 | 313 |  |  |
|  |  |  |  |  |  |  |  |
| **Absolute risk** (per 1000 person years) | 4.667  (4.310, 4.996) | |  | 4.323  (3.869, 4.829) | |  |  |
|  |  |  |  |  |  |  |  |
| **BMI at age 20 years (kg/m^2^)** |  |  |  |  |  |  |  |
| < 18.5 | 2349 | 81 | 1.166  (0.891, 1.525) | 1060 | 30 | 1.358  (0.878, 2.070) |  |
| 18.5 to 22.4 | 17861 | 478 | 1.000 | 8204 | 168 | 1.000 |  |
| 22.5 to 24.9 | 8039 | 188 | 0.874  (0.727, 1.050) | 3486 | 79 | 1.237  (0.932, 1.641) |  |
| 25.0 to 29.9 | 3326 | 71 | 0.797  (0.602, 1.056) | 1577 | 34 | 1.082  (0.719, 1.629) |  |
| ≥ 30 | 703 | 11 | 0.505  (0.239, 1.068) | 437 | 2 | 0.156  (0.022, 1.118) |  |
| Per SD (3.23 kg/m^2^) |  |  | 0.866  (0.794, 0.945)  P = 0.001 |  |  | 0.919  (0.800, 1.056)  P = 0.234 | P = 0.354 |
|  |  |  |  |  |  |  |  |
|  |  |  | **Model D** |  |  | **Model D** |  |
|  | **n** | **cancers** | **HR**  **(95% CIs)** | **n** | **cancers** | **HR**  **(95% CIs)** |  |
|  |  |  |  |  |  |  |  |
| **BMI at age 20 years (kg/m^2^)** |  |  |  |  |  |  |  |
| < 18.5 | 2349 | 81 | 1.238  (0.946, 1.622) | 1060 | 30 | 1.362  (0.885, 2.097) |  |
| 18.5 to 22.4 | 17861 | 478 | 1.000 | 8204 | 168 | 1.000 |  |
| 22.5 to 24.9 | 8039 | 188 | 0.777  (0.643, 0.939) | 3486 | 79 | 1.215  (0.903, 1.634) |  |
| 25.0 to 29.9 | 3326 | 71 | 0.626  (0.465, 0.842) | 1577 | 34 | 1.042  (0.666, 1.632) |  |
| ≥ 30 | 703 | 11 | 0.333  (0.155, 0.718) | 437 | 2 | 0.146  (0.020, 1.075) |  |
| Per SD (3.23 kg/m^2^) |  |  | 0.774  (0.703, 0.852)  P < 0.001 |  |  | 0.893  (0.757, 1.053)  P = 0.178 | P = 0.470 |
|  |  |  |  |  |  |  |  |

BMI: body mass index. CI: confidence interval.

*Cox model with attained age as timescale.

† Weight changes (absolute and relative) are across the time from recall at age 20 years to cohort entry.

Model B: adjusted for age at study entry, height, age at menarche, race (White, Asian, Black, Jewish, Others, missing), hormonal replacement therapy (ever/never user), statin (ever/never user), alcohol use (yes/ no/ missing), any exercise (yes/ no/ missing), number of children, age at first pregnancy, hysterectomy, number of ovaries removed.

Model D: as for model B, plus adjustment for BMI at cohort entry.

**Table S10 Hazard ratios* and 95% CIs for all new breast cancers for BMI and absolute weight changes in 47042 women in PROCAS (2009-2015), stratified by age categories at entry. Tests for interactions by exposure*attained age to assess for survivor bias**

|  | **Age category**  **44.0 to 53.9 years** | **Age category**  **54.0 to 59.9 years** | **Age category**  **≥ 60.0 years** |
| --- | --- | --- | --- |
|  | **HR**  **(95% CIs)** | **HR**  **(95% CIs)** | **HR**  **(95% CIs)** |
|  |  |  |  |
| No. of women | 16,116 | 11,222 | 19,704 |
|  |  |  |  |
| **Body mass index at study entry (kg/m^2^)** |  |  |  |
| Interaction term: BMI*age | 0.999  (0.988, 1.010)  P = 0.857 | 1.001  (0.987, 1.015)  P = 0.850 | 0.998  (0.993, 1.003)  P = 0.500 |
|  |  |  |  |
|  |  |  |  |
| **Weight change (absolute – kg)** |  |  |  |
| Interaction term: weight_change*age | 0.999  (0.994, 1.004)  P = 0.772 | 1.003  (0.998, 1.010)  P = 0.236 | 1.000  (0.998, 1.003)  P = 0.664 |
|  |  |  |  |

BMI: body mass index. CI: confidence interval.

*Cox model with attained age as timescale.

All models: adjusted for age at study entry, height, age at menarche, race (White, Asian, Black, Jewish, Others, missing), hormonal replacement therapy (ever/never user), statin (ever/never user), alcohol use (yes/ no/ missing), any exercise (yes/ no/ missing), number of children, age at first pregnancy, hysterectomy, number of ovaries removed, menopausal status (post-menopausal versus pre- and peri-menopausal), and BMI at age 20 years

**References**

**1.** Keum N, Greenwood DC, Lee DH, et al. Adult weight gain and adiposity-related cancers: a dose-response meta-analysis of prospective observational studies. *J Natl Cancer Inst.* Feb 2015;107(2).

**2.** World Cancer Research Fund/ American Institute for Cancer Research. Diet, Nutrition, Physical Activity amd Cancer: a Global Perspective. Continuous Update Project Expert Report 2018 dietandcancerreport.org

**3.** Feigelson HS, Jonas CR, Teras LR, Thun MJ, Calle EE. Weight gain, body mass index, hormone replacement therapy, and postmenopausal breast cancer in a large prospective study. *Cancer Epidemiol Biomarkers Prev.* Feb 2004;13(2):220-224.

**4.** Lahmann PH, Schulz M, Hoffmann K, et al. Long-term weight change and breast cancer risk: the European prospective investigation into cancer and nutrition (EPIC). *Br J Cancer.* Sep 5 2005;93(5):582-589.

**5.** Eliassen AH, Colditz GA, Rosner B, Willett WC, Hankinson SE. Adult weight change and risk of postmenopausal breast cancer. *JAMA.* Jul 12 2006;296(2):193-201.

**6.** Palmer JR, Adams-Campbell LL, Boggs DA, Wise LA, Rosenberg L. A prospective study of body size and breast cancer in black women. *Cancer Epidemiol Biomarkers Prev.* Sep 2007;16(9):1795-1802.

**7.** Ahn J, Schatzkin A, Lacey JV, Jr., et al. Adiposity, adult weight change, and postmenopausal breast cancer risk. *Arch Intern Med.* Oct 22 2007;167(19):2091-2102.

**8.** Kawai M, Minami Y, Kuriyama S, et al. Adiposity, adult weight change and breast cancer risk in postmenopausal Japanese women: the Miyagi Cohort Study. *Br J Cancer.* Oct 26 2010;103(9):1443-1447.

**9.** Alsaker MD, Janszky I, Opdahl S, Vatten LJ, Romundstad PR. Weight change in adulthood and risk of postmenopausal breast cancer: the HUNT study of Norway. *Br J Cancer.* Sep 3 2013;109(5):1310-1317.

**10.** Catsburg C, Kirsh VA, Soskolne CL, et al. Associations between anthropometric characteristics, physical activity, and breast cancer risk in a Canadian cohort. *Breast Cancer Res Treat.* Jun 2014;145(2):545-552.

**11.** Rosner B, Eliassen AH, Toriola AT, et al. Weight and weight changes in early adulthood and later breast cancer risk. *Int J Cancer.* May 1 2017;140(9):2003-2014.

**12.** Cole TJ, Freeman JV, Preece MA. Body mass index reference curves for the UK, 1990. *Arch Dis Child.* Jul 1995;73(1):25-29.
